# The co-occurrence of substance misuse, domestic abuse, and child maltreatment: Can Family Drug and Alcohol Courts play a part?

**DOI:** 10.3389/fpsyt.2022.989813

**Published:** 2022-10-19

**Authors:** Judith Harwin, Charlotte Barlow

**Affiliations:** ^1^Centre for Child and Family Justice Research, Law School, Lancaster University, Lancaster, United Kingdom; ^2^School of Justice, University of Central Lancashire, Preston, United Kingdom

**Keywords:** domestic abuse and violence, child protection, care proceedings, substance misuse, Family Drug and Alcohol Courts, family justice, criminal justice, family policy and law

## Abstract

This review article focuses on the inter-relationship between substance misuse, domestic abuse, and child maltreatment, especially in the context of care (child protection) proceedings. It reviews what is known about the prevalence and impact of co-occurring domestic abuse and substance misuse on adult and child victims, and the response of criminal and family law and intervention programmes in supporting families to address these problems holistically. Special attention is paid to the role of Family Drug and Alcohol Courts (FDACs), a radical problem-solving approach to care proceedings, which provide integrated interventions to the range of co-occurring problems that trigger the proceedings. Despite clear evidence of the greater harm to children when exposed to these two parental difficulties, the review has found a lack of systematic information on the prevalence of co-occurrence and a lack of effective integrated interventions, including within care proceedings. It argues that the FDAC approach is well suited to respond to co-occurring substance misuse and domestic abuse in care proceedings and it has the potential to break down silos across sectors. However, in the absence of empirical evidence, this premise would need testing. A particular focus of the review has been on efforts to overcome silos in practice, law and policy. Promising initiatives are described in criminal and family law to improve the response to domestic abuse that build on the Domestic Abuse Act 2021, the first dedicated domestic abuse legislation in England and Wales. All of them are based on problem-solving approaches used in other jurisdictions. Despite these initiatives, the review concludes that there remain significant barriers to effectively align law, policy and practice to ensure that domestic abuse strategy recognizes and responds to the overlaps with substance misuse.

## Introduction

In 1847, the British illustrator George Cruikshank, published his series “The bottle” (1). It had huge impact, charting the corrosive inter-relationship between paternal alcohol misuse and domestic abuse (hereafter referred to as DA). The images and messages were stark, demonstrating what Cruikshank saw as an inevitable and unstoppable decline that ended with the wife being killed by her husband, the death of one of their children, and the father ending up in an asylum. Cruikshank's remedy was the temperance movement, which urged total abstinence. At the time there were no treatments available for substance misuse and child abuse was a private matter. In this regard, Lord Shaftesbury, best known for his reform to child labor, declared in 1875 that “The evils you state are enormous and indisputable, but they are of so private, internal and domestic a nature as to be beyond the reach of legislation and the subject would not, I think, be entertained in either house of Parliament” (2). This cameo captures many of the key elements to be explored in this review- the role of legislative support for intervention in the family when DA and substance misuse coexist; the availability of appropriate family interventions, and the extent to which they are supported by public policy and effective practice.

In this article we examine the inter-relationship between substance misuse, DA and child maltreatment with special attention to the family courts and consider what role they can play in addressing these problems holistically. Particular attention is paid to the potential of family drug treatment courts (FDTCs)^1^, known in England and Wales as Family Drug and Alcohol Courts (FDACs), to address co-occurring DA and substance misuse in care proceedings. Care proceedings have a child protection function and determine whether the child can remain with the birth parent or needs alternative permanent care. Unlike ordinary care proceedings, FDACs are designed to address parents' multiple problems *holistically within* the care proceedings, as well as adjudicating on the type of legal order, if any, is needed, and placement arrangements. FDTCs originated in the USA and have been adopted by other adversarial child protection systems, including Victoria State, Australia, and England and Wales, and they have some success in family reunification, at least in the short term. They are a type of problem-solving court which are also widely used in criminal justice in the US to address issues such as an individual's drug offending. Central to all problem-solving courts is the premise that without treating the underlying problems, legal interventions will be ineffective or indeed, anti-therapeutic (3, 4).

The purpose of this article is to bring together in a single paper a broad-ranging and scattered literature by means of a comprehensive review that addresses five main questions:

What is known about:

1. The inter-relationship between co-occurring DA, substance misuse and child maltreatment.
2. The nature and scale of co-occurrence of substance misuse and DA.
3. The response to the co-occurrence of DA and substance misuse within the family and criminal justice system, and in treatment interventions.
4. The potential of Family Drug and Alcohol Courts to play a part in the response to co-occurring DA and substance misuse.
5. What are the implications and recommendations from this review for policy, practice and research?

The review mainly focuses on the experience of England to illustrate the issues, many of which are relevant to an international audience insofar as the co-occurrence of substance misuse and DA affect many societies. With new legislation on DA introduced in 2021 and pilot problem-solving courts being trialed in criminal and family law for substance misuse and/or DA, England is particularly interesting to study.

To address the questions outlined above, the methodology draws on the principles of scoping reviews (5). It therefore addresses a range of broad topics rather than providing in-depth coverage of specific questions, or consideration of the quality of the studies. As is also the case in scoping reviews, key informant sources have been used as well as drawing on the authors' own knowledge and research.

Finally, with regard to terminology, for simplicity, we use “substance misuse” to cover alcohol misuse and drug misuse, and to denote that it is causing harm. Unless specifically referring to studies of intimate partner violence, we use the umbrella term “domestic abuse.” When we refer to child maltreatment, it covers all types of abuse and neglect experienced by children. Government guidance classifies witnessing DA as a form of emotional abuse (6) and the Domestic Abuse Act 2021 specifies that children are victims in their own right if they “see, hear or experience the effects of abuse,” linked to a parent or relative's behavior.

## What is known about the inter-relationship between parental substance misuse, domestic abuse, and child maltreatment?

There is a consensus that the cumulative risk to children increases when parental substance misuse and DA co-exist (7–11), particularly when it is the perpetrator who is engaged in substance misuse (12–15). It increases the risk of serious injury or death (16). Co-occurrence of substance misuse and DA affects parenting capacity and increases the risk of abuse, neglect, and subsequent child removal by children's services. It is also associated with return to care and breakdown of reunification (11, 16, 17).

It is easy to understand why risks to the child increase when DA is accompanied by parental substance misuse. As the definition in the Domestic Abuse Act 2021 makes clear, no area of the adult victim's life is unaffected, making parenting more stressful and coping harder. The impacts fall disproportionately on women because men are much more likely to perpetrate DA than women. Coercive and controlling behavior, in particular, is almost exclusively perpetrated by men against women and is a course of conduct aimed at dominating and controlling another (usually an intimate partner) (18, 19). The impacts on adult victims are well documented. DA is associated with depression, suicidal ideation and self-harm, anxiety disorders, phobias, eating disorders, post-traumatic stress disorder and physical injury (12, 20). The more severe the experience of physical and sexual violence, the more likely it is to be associated with substance misuse by the victim, as well as with homelessness, disability, and poverty (20, 21). When a child's mother misuses substances, which itself is associated with inconsistent or harsh parenting (22) and increased risk of child maltreatment (23), DA compounds the risk of failure to protect the child and response to basic emotional needs.

For children, the harms of DA include emotional and behavioral problems, psychological disorders, truancy, bedwetting, mental health difficulties, physical injury, self-harm and use of alcohol and drugs (7, 9, 11, 20, 24–28). These harms can persist into adulthood. Witnessing domestic violence between parents can increase the risk of heart disease, stroke, substance misuse, depression, and suicide attempts (29). Barnett [(24), p. 15] concluded from her literature review for the Ministry of Justice that “living with coercive control can have the same cumulative impact on children as it does on adult victim/survivors, which may contribute to emotional and behavioral problems in children.” Barnett also suggests that it would be more accurate to describe children as experiencing DA rather than being “witnesses” or “exposed to DA,” as they are victims in their own right. When the child is also exposed to parental substance misuse as well as DA, risks of harm are compounded.

More attention has been paid to investigating the association between alcohol misuse and DA than between illegal drug misuse and DA (14, 30, 31). The association between alcohol misuse and DA has been upheld globally, with one third of women and children affected (32). Whilst there is little evidence to show a causal connection (20, 30, 32, 33), the risks of DA increase when it co-exists with alcohol misuse, as does the severity of physical injuries (33, 34). Rates of physical or sexual violence perpetration are four times higher in men undergoing substance misuse treatment than in the general population (35). Gilchrist and Hegarty (35) also found that among a sample of men in substance use treatment in England, the majority had perpetrated intimate partner violence during their current or most recent relationship.

The association between alcohol misuse and DA is complex and multifactorial (7, 20, 30, 31, 36). For perpetrators, contributing factors can include the disinhibiting effects of alcohol and its impacts on cognitive processes that can result in perceptual biases such as “hostile attribution biases” (32). The risk of intergenerational transmission of patterns of violence and misuse of alcohol can also increase when perpetrators have themselves experienced childhood adversity, especially if it involved violence and abuse (37, 38). Furthermore, female victim-survivors may use alcohol to cope with DA (9, 20, 39, 40) or other traumas, which, as already noted, has been associated with reduced attention to children's needs (7, 30, 41). Perpetrators may use alcohol as a way of controlling their partners (20). Interactions between intimate violent partners are particularly complex where both partners are substance dependent (42). They may threaten to report the child's mother to children's social care services to silence her. Fear of child removal by the state is one of the most potent ways in which domestic abuse can play out in families, causing delay in seeking help for the adult and child victim and it is exacerbated by maternal substance misuse.

The evidence on the increased risk of child neglect and abuse when DA and substance misuse co-occur is strong. Moreover, child maltreatment also increases the risk of experiencing DA in adult life (43) so there is a transgenerational link. The practice and policy implications are clear. Victim survivors, perpetrators and children need to be able to access targeted interventions which can respond to both issues in an integrated and timely way.

### What is known about the nature and scale of co-occurrence of substance misuse and domestic abuse?

There is little systematic information on the prevalence of co-occurring DA and substance misuse, but it is recognized to be an international problem (31, 36, 40, 44, 45). In England and Wales national evidence is limited on the co-occurrence of substance misuse and DA, although secondary data analysis of the Adult Psychiatric Morbidity Survey (APMS) and the Crime Survey for England and Wales (40, 46) both uphold the association. However, the Crime Survey excludes coercive and controlling behavior^2^ and therefore it is likely to underestimate the scale and nature of the problem^3^. Moreover, the association is complex. Analysis of the APMS found the severity of physical and sexual violence increases the likelihood of victims' misuse of drugs and alcohol (21). These victims, mainly women, were more than twice as likely to misuse alcohol, and eight times more likely to be drug dependent than women who had little experience of violence and abuse. Focusing on a comparison of mothers and women without children, a study of the electronic health records of women attending addiction services found that mothers were almost five times more likely to have experienced DA over their lifetime than women without children (47). Mothers with children in care were more likely to report DA than those who were still living with their children. Co-occurring DA and parental substance misuse featured most prominently in a longitudinal study when the parents were part of a small sub-group described as “poly-diversity” (26) who had the most severe problems.

As regards children, in the view of the Children's Commissioner there is a dearth of data on co-occurrence, and it is not a straightforward problem to measure (48). However, it is a sizeable problem. In a typical class of 30 children, four will live in a household with DA *and* parental substance misuse *or* severe parental mental ill health (49). There is also a troubling lack of data on the prevalence of co-occurring DA and substance misuse in children's services and in the family courts because this information is not routinely collected in this way. These issues with data extend to the police recording of DA offenses, as the Police National Computer (PNC) cannot link DA and alcohol or substance misuse, meaning that the extent of co-occurrence is unknown.

Taken together, the available evidence suggests that there is a shortage of reliable information for policy-makers, service providers and researchers on which to plan and deliver services that can respond in an integrated way to co-occurring substance misuse and DA.

### What is known about the scale of domestic abuse as a single issue?

The scale of DA as a single issue in England and Wales is formidable. The Crime Survey for England and Wales estimated that 2.3 million adults aged between 16 and 74 experienced DA year ending March 2020 (20). Over the same period an estimated 5.5% of adults aged 16 to 74 experienced DA in the year prior to the survey (50). The victim was female in 73% of DA related crimes. Over 40% of victims have at least one child under the age of 16 living in their household (20). According to the Children's Commissioner, in a typical class of 30 children, two will live in a household where DA is present (49).

The impact of DA on children's social care is considerable. This is demonstrated by a range of figures brought together by the Independent Review of Children's Social Care, set up in fulfillment of the Government's manifesto to review the children's social care system (51). Violence between parents is the most common factor identified at the end of social work assessments for children in need^4^ while DA (42%), parental mental ill-health (28%), drug (24%) and alcohol (18%) use are frequent factors in incidents involving serious injury or death, especially for children aged under one. DA was present in 64% of families where child neglect was the issue in serious case reviews which are carried out when children have died or been seriously injured (16).

DA is also a major issue in private and public law proceedings. Allegations or findings of DA are present in between 49%−62% of private law family proceedings where the issue is about who the child will live, or have contact with (52, 53). DA also plays a prominent part in care proceedings and in recurrent care proceedings. A national study of all care proceedings between 2007 and 2016 (175,280 children) found that DA was one of the triggers to the proceedings for more than half (56%) of the children (54). Moreover, DA also significantly increased the probability of maltreatment for children returned home on a supervision order from 18% to 55% in the 4-year follow-up. The importance of DA also emerged very strongly from a national study of mothers who have recurrent care proceedings, for the same or a new child. Of the 11, 191 mothers involved in recurrent care proceedings between 2007/07 and 2015/16, DA was a key concern in 65% of the cases (55). These mothers were also highly likely to have had a parent with DA issues, underlining the destructive transgenerational continuities. As regards fathers involved in recurrent care proceedings, a national study found that the most common child welfare concerns were substance misuse, DA and poor mental health (56). The repercussions of these issues are significant. Data from care proceedings and child protection cases indicate that up to 60% of children who are fostered, adopted or in other out of home care arrangements may have been victims of DA (16). In short, DA significantly impacts on the family court and plays a prominent role in child separation from parental care, intergenerational transmission, substitute care services and failed reunification (17). These are deeply troubling statistics, but they do not present the full picture because of limitations in what data can be, and is, collected. Crucially, each of these three national studies of care proceedings found that substance misuse was a prominent concern, amongst issues such as mental health difficulties, relationship problems, lack of social supports, deprivation, housing problems and non-engagement with services.

However, there is a lack of literature available which specifically explores the co-occurrence of DA, substance misuse and child maltreatment within the context of children's social care and care proceedings. This severely limits understand of the issues and the possibility of developing tailor-made services that recognize the interconnections and complexity of the issues. The largest, and most recent profiling of cases involving parental substance misuse in an English local authority, found that of the 299 children referred to children's social care, 42.8% were also exposed to DA (57). Risk, or actual abuse and neglect, were the most frequent reasons for referral. An earlier study of all children referred to children's services in four authorities found a complicated relationship between parental substance misuse, DA and child outcomes (58). Illegal drug misuse was more likely to result in the permanent removal of infants at birth than alcohol misuse following care proceedings. By contrast, toddlers and older children affected by parental alcohol misuse were more likely to remain at home with their birth parents and to be subsequently exposed or re-exposed to DA. As a result, their outcomes at follow-up were worse than for infants removed at birth. Not only does this suggest that alcohol misuse was perceived by social workers to be less harmful than drugs, but the social workers also revealed that it was particularly difficult to work with parents on their alcohol problems if one of the parents, usually male, was violent, as “professional fear (for their own safety) kicked in.”

Some of the studies on care proceedings also indicate the possibility of co-occurrence but as in children's social care, data on prevalence is often presented separately for each issue. For example, a study of court records of 386 care proceedings cases (682 children) found that maternal DA (51.1%) was ranked amongst the most common problems, with drugs featuring in 38.6% of the sample, and alcohol abuse in 25.3% (59). As already noted, DA also features significantly amongst mothers who return to court for further care proceedings, either for the same or a new child (55). DA and substance misuse are also linked to non-engagement with children's social care, and this may help explain why risk of recurrent proceedings increased when DA was associated with substance misuse (55, 59). Furthermore, the financial costs of DA are significant, with the annual cost in the UK equating to ~£66 billion (60, 61).

These interrelationships between substance misuse, DA and child maltreatment, as already noted, have tended to be analyzed as correlates rather than as interrelated and they are viewed as part of a widely accepted picture of a “toxic trio” comprising mental health difficulties as the third element. (The other two are substance misuse and DA). That approach has only recently been challenged (62). From a systematic review of twenty studies, the authors have argued that evidence of this association lacks rigor. This is because of a lack of comparison groups, failure to look at wider contextual factors such as child age, ethnicity and socio-economic status, the quality of interventions, and consideration of theoretical explanations of the nature of these inter-relationships. Skinner and colleagues call for a change in the discourse, which they argue has had a disproportionate and unhealthy influence on the child protection and family justice system.

A starting point would be to make use of national largescale administrative datasets, such as those held by Cafcass^5^ and the Ministry of Justice, for all children subject to care proceedings, to investigate the prevalence of co-occurring parental substance misuse and DA, and their relative importance in “significant harm”^6^ and the initiation of proceedings. However, at present, court records do not have any flags for DA, substance misuse and mental health problems, or agreed definitions of each issue. This means that we are dependent on research studies to provide estimates of the nature and strength of the associations between the different parental issues and their impact on child harm, and the likelihood of triggering care proceedings. One promising approach to achieving a better understanding of how substance misuse and DA may cluster, and the possible different permutations, is to use latent class analysis (LCA). LCA is a statistical procedure which divides groups of people with common characteristics into clusters, or sub-groups (63). It enables fine-grained information on patterns of associations, which can then be used to provide better targeted interventions. For example, using LCA, Broadhurst et al. (55) discovered in their study of recurrent care proceedings, that DA was found in different combinations, some with, and others without substance misuse. They concluded that identification of these sub-groups provided a much better understanding of the issues than the broad term “toxic trio” and could help generate interventions tailored to the needs of the particular sub-group.

Drawing together the available evidence, we can conclude that DA, child safeguarding and substance misuse are related issues because of their collective damaging effects on children and women (27, 36). This is not to say that men are not victims of DA or affected by their female partner's substance misuse. But the risks are higher for women and children. The increased recognition of the harms associated with DA is evidenced by the flurry of policy development aiming to tackle the issue.

## The response to domestic abuse and substance misuse in criminal and family law and policy

### Criminal law

Many policy interventions to date in England and Wales related to DA and associated issues such as substance misuse, have focussed on criminal law and justice interventions. Over the last decade there has been an expansion of DA law as awareness of the problem has grown and recognition that the state must intervene in what for many years was seen as a private problem. It has included Section 76 of the Serious Crime Act 2015, criminalizing coercive and controlling behaviors within intimate or familial relationships, and the more recent Domestic Abuse Act 2021. This is the first dedicated legislation on DA in England and Wales and it contains many welcome features to better protect victims and their children, including the creation of a Domestic Abuse Commissioner. For the first time the legislation creates a statutory definition of DA in England and Wales, including the addition of economic/ financial abuse. Non-fatal strangulation and suffocation, which increases the risk of being killed by a perpetrator sevenfold is also a new offense under the Act and punishable by up to 5 years in prison^7^. Notably however, the Domestic Abuse Act 2021 does not create a new offense of domestic abuse. It provides however, a new civil Domestic Abuse Protection Notice/ Order (DAPN/DAPO) and places the Domestic Violence Disclosure Scheme (DVDS) on a statutory footing. DAPNs are intended to give victim-survivors and children an additional protection measure immediately after a DA incident, providing breathing space or “space for action” (64). DAPOs will be more flexible in duration (20) and, as well as specifying prohibitions, will have a positive function, for example, requiring perpetrators to attend behavioral change programmes or substance misuse programme. Breach will be a criminal offense but with the option of a civil contempt of court that takes into account public interest and the victim's views. Crucially, in terms of the potential to break down silos, it will be possible for the family, criminal and civil court to make a DAPO of their own volition, even if the issue is not DA related. They are to be piloted before being introduced across the UK.

The DVDS, better known as Clare's Law, gives police the right to disclose information about past DA convictions to the public or to a current partner (65). However, it has been argued that DAPOs can often lead to the downgrading of DA in criminal justice terms. The dual regime of civil and criminal protective orders has led to an increase in charges for breaches of protective orders after an allegation of DA. Whilst this is to be welcomed, Bates and Hester (66) express concern that an increase in charges for breach, may obscure a corresponding drop in charges for substantive offenses that are charged for DA. This therefore limits their potential capacity to keep women and children safe. Furthermore, DVDS comprises two aspects, “a right to ask” (an application can be made by any member of the public to apply to the police for information about whether a person has a history of domestic abuse) and a “right to know” (the police act proactively to disclose information to protect a potential “high-risk” victim from harm from their partner if that partner has a known history of abuse). However, Barlow et al. (67) identified that victim-survivors can often feel disempowered and responsibilised by such schemes and questioned their protective and preventative value.

More positively, the Domestic Abuse Act 2021 recognizes children as victims in their own right and places new duties upon local authorities to assist with housing to ensure the safety of the child and parent and to provide support to victims and their children fleeing an abusive relationship. This enhanced statutory duty upon local authorities is to be welcomed as DA victims and their children are at high risk of housing instability and homelessness (68). Moreover, the types of specified support are wide-ranging. They include counseling and therapy, housing-related advice and support, communicating with other health and social care providers, specialist support for victims with complex needs and/or protected characteristics and helping victims to recognize the signs of abusive relationships to prevent re-victimization^8^. However, the duty is limited to victims and their children who have fled their home and are living in refuges and other specified types of accommodation and to authorities classified as “tier 1”^9^. Moreover, children's services are not a housing provider and there is a serious shortage of suitable accommodation (69).

A further example of this emphasis on criminal justice approaches is the introduction of Specialist Domestic Violence Courts (SDVCs) across England and Wales approximately two decades ago. A key rationale for their introduction was to reduce victim withdrawal/ retraction from the criminal justice process, a common feature of many DA cases (70), and to improve victim satisfaction (71). Basic features of SDVCs include identifying DA cases and thereafter “fast tracking” them, having an advocate present to support victims, and a specialist police officer to provide information in court. However, despite positive intentions, SDVCs face notable issues. First, SDVCs in England and Wales lack key powers and resources, including the option to monitor offenders in the community. Second, Cook et al.'s (71) evaluation suggested that the establishment of SDVCs was not necessarily leading to fewer victim retractions, despite being a key rationale for their introduction. Third, despite DA continuing to rise in England and Wales, conviction rates continue to drop (72). This means that at a time when more victim-survivors are reporting DA, the justice system is securing fewer convictions (73). Finally, although SDVCs signpost victim-survivors to other services, they do not provide interventions for DA and substance misuse holistically within the context of the court intervention itself (73). This is despite the fact that Cook et al. (71) found that victims were more likely to retract when the defendant had committed the offense following alcohol use. This highlights issues with silo-working. In sum, although SDVCs have the potential to improve victim satisfaction, they do not provide a holistic, whole family response to effectively deal with co-occurring DA and substance misuse in their current form.

Relatedly, the Ministry of Justice (74) is developing up to five criminal problem-solving court pilots, to address (i) substance misuse offenders facing custody (ii) perpetrators of DA and (iii) female offending. The first two problem solving courts for drugs and alcohol are to be piloted as part of the government's 10-year drug strategy *From Harm to Hope* while the court for female offenders with complex needs will also include measures to address substance misuse (75). Problem solving courts provide an alternative to traditional courts by targeting a specific client group, offering multidisciplinary assistance within the court process, judicial monitoring, a transparent, procedurally fair process and in the criminal context, “graduated incentives and sanctions” (76, 77). Problem-solving criminal courts originated in the USA, and they are used in a number of countries such as New Zealand, Australia, Canada, Scotland and Norway. Evaluation studies show that re-arrest and re-offending rates are lower than those of matched drug offenders going through ordinary criminal court and there are associated cost savings (78–80). These new Ministry of Justice pilot courts are to be welcomed, but it is noteworthy that they are being set up as single-issue concerns, rather than as dealing with interconnected and overlapping problems.

However, despite decades of the creation of more criminal laws and criminal justice policies related to DA [what Goodmark (81) terms “the criminalization thesis”], two women a week are still killed by a current or former partner in the UK (72) and the numbers of people experiencing DA remains persistently stubborn. The question therefore remains as to what this flurry of legislation has achieved for safeguarding victim-survivors and their children? Furthermore, much work related to safeguarding victim-survivors and their children more broadly has been developed in silos, with little emphasis on holistic or whole systems approaches (82). Such issues also translate to practice in family law.

### Family law

The Adoption and Children Act 2002 was the first to recognize in statute that witnessing DA could cause actual or likely significant harm. It widened the definition of “significant harm” set out in the Children Act 1989 to include, “impairment suffered from seeing or hearing the ill-treatment of another”^10^. Despite this legal landmark, progress in responding to DA in the family justice arena has been slow and patchy. The highly influential Ministry of Justice *Harm Panel Report* (83) shone a spotlight on the inadequate professional response to DA in private law proceedings and put forward a raft of recommendations for wide-ranging policy, system and practice changes. Many of the barriers it cited were well known. They included false accusations of parental alienation (84), systems abuse (85, 86), and perpetrators using the family court as a site of coercive control (86). In addition, and crucially for the present review, the Panel report highlighted the obstacles to reform resulting from working in silos with poor coordination with other courts and organizations dealing with DA; an adversarial process; a lack of resources; insufficient attention to assessment of risk and child safeguarding and an inability to hear children's voices.

One way in which the issue of silos is to be addressed is via the creation of pilot Integrated Domestic Abuse Courts (IDACS). The intention is to trial a “one family, one judge” approach which, in some family and criminal proceedings involving DA, are to be heard by the same judge who is authorized to hear both family and criminal matters (74). The aim is to reduce silos between the two jurisdictions and thereby prevent victims being retraumatised by having to present their evidence more than once. At the same time, to promote a less adversarial and more investigative process, two pilots in North Wales and Bournemouth (87, 88) are being set up to deal with DA allegations in child arrangement disputes in private family law cases when parents are separated, especially when coercive control is a key issue. They will run until 2024.

There are a variety of IDACs internationally based on the “one family, one judge” approach but evidence of their effectiveness is considered weak (89). The most ambitious IDACs have the case heard by a judge who is authorized to hear criminal, family (including child protection) and potentially civil proceedings, as for example in New York. Little detail is available at present on the English schemes, but important considerations of their potential contribution include the availability of judges authorized to hear both family and criminal cases and families' reactions to the dual mandate. Furthermore, there is limited evidence to suggest that IDACs will provide the holistic approach required to deal with co-occurring substance misuse and DA, to which we shall return later in the review.

There has been no equivalent to the *Harm Panel Report* in public law proceedings. Moreover, the crossover between private and public child proceedings has received little attention (90). More broadly, family justice policy has been insufficiently integrated with criminal policy, despite the obvious overlaps. For example, in response to the Home Office's consultation on the development of its domestic abuse strategy, an informal alliance of organizations (91) called for a discrete section on the family court in the strategy to signal its important role. Much existing work with perpetrators, particularly court sanctioned programmes, have also faced criticism, both in terms of a lack of reduction in DA (particularly coercive control) and a lack of integrated support for perpetrators (92). The *Harm Panel Report* called for a review of DA perpetrator programmes to ensure they address child safety and welfare needs when DA is an issue (83).

The response to DA and substance misuse in care proceedings has scarcely begun to be charted, though studies of parental perspectives have charted the profoundly damaging impact of the adversarial process on mental wellbeing since 1998 (93, 94). More than two decades later, parents are describing the same issues in their experiences of care proceedings (90). Despite having their children returned to their care, the parents at the end of the court case described the care proceedings as “inhumane” and “belittling” and felt that they were made to feel like “criminals.” In their view, the court process exacerbated their mental health difficulties and stress levels and revictimized them, undermining their perceptions of access to justice. When asked to identify how care proceedings could be improved, parents prioritized the need for a more effective response by the court to DA. With evidence to show that one in three or four mothers nationally risk return to court for further care proceedings within seven years (55), there is a vital need to find alternative ways for the courts to respond to DA and parental substance misuse, and more specifically in an integrated way.

Hester (82) suggests that despite the positive development of much work to reduce intimate partner violence, there remain difficulties and frustrations inhibiting safe outcomes for victim-survivors of DA and their children. She identifies some of the disconnections, tensions, and contradictions evident in professional discourse and practice across three different professional practice arenas: domestic violence (all the agencies dealing with victims and perpetrators), child protection (social work) and child contact (family courts). She concludes that these arenas are especially difficult to bring together in a cohesive and coordinated way because each is essentially “on a different planet.” She asserts that such a fragmented “three planet model” precludes effective responses to IPV, and indeed may result in outcomes likely to be counter-productive for individuals interacting with them. This fragmented approach is even more evident when substance misuse and mental health are added into the mix, with a lack of joined up working evident in the support provision provided in such contexts. Thus Hester (82) argues for systemic change, orientated toward a cohesive, coordinated, and unified approach across these planets, centring gender both as a feature of DA and associated issues such as substance misuse, and as a feature of service delivery to ensure more effective policy intervention. Similar issues with siloed working have been identified in treatment offerings for DA and substance misuse (30, 44).

## Interventions for co-occurring substance misuse and domestic abuse

The international evidence indicates a lack of effective interventions for co-occurring substance misuse and DA (35, 95–99). As both issues are over-represented in households in England where parents are on benefits, the Department of Work and Pensions commissioned a review to identify interventions addressing both substance misuse and “parental conflict”^11^ (14). The authors found few. Moreover, they questioned the effectiveness of current models of treatment in a family justice or children's social care setting. They found that psychoeducational and cognitive behavioral treatment programmes predominate, especially within children's social care and the family court, with two thirds of referrals to perpetrator programme referrals coming from Cafcass or children's social care (100). In their view, reliance on programme completion as a means of reducing risk, given the limited evidence base, is a cause for concern. The Independent Review of Children's Social Care also highlighted the lack of research evidence on effective interventions for substance misuse and DA in children's social care settings (51). There is a dearth of community based early intervention integrated services, or services targeted at children or fathers (95, 98, 101). Stover (99) noted that recidivism in most perpetrator and partner-focussed treatments was ~30% within 6 months, regardless of the model, and more recently focused on the need for effective programmes to work with perpetrators in their capacity of fathers (99). Few treatment interventions have been evaluated (102).

As professional awareness has grown regarding the destructive impacts of co-occurring substance misuse and DA, new approaches are being piloted and evaluated in community settings. For perpetrators, a 60-month programme called “Advance” (96) is testing a 14-week intervention that addresses the substance misuse and DA in an integrated fashion in the health sector. At present, this group of adults rarely access treatment, despite the higher rate of partner abuse. Preliminary results are encouraging. The feasibility study found that, compared to men who received a substance misuse intervention only, men who also received the Advance Programme intervention, reported higher levels of satisfaction, as did the staff (103). In children's social care services, a community programme, Family Safeguarding Hertfordshire (FSH) has aimed for systemic reform and it has been adopted by other local authorities due to encouraging results. The intervention comprises a partnership that includes the police, health (including mental health), probation and substance misuse services. Key elements include specialist workers with DA, substance misuse and mental health expertise joining teams and training in motivational interviewing. The evaluation found that the inclusion of adult specialist workers in the children and family teams were particularly beneficial, bringing multidisciplinary expertise to the families (104). The families were positive about the help they received and fewer days were spent in care than before FSH was introduced. In Australia, a protocol has been approved to test the effectiveness of an integrated approach to tackling co-occurring substance misuse and DA. This protocol, known as Kody, is also a whole family approach and builds on the existing evidence of the Kody programme (95).

The search for effective integrated interventions is essential and potentially stands to reduce the very high costs of programmes that fail or do not reach the target groups. So the new initiatives outlined above are encouraging, but they are few and far between. Furthermore, as previously noted, there is widespread agreement in the international literature that most services are delivered in silos and that an integrated approach is needed, which addresses whole family needs holistically (30, 33, 52, 97, 102).

The real question is why there are so few integrated services available, given consistent evidence on the strength of the association between DA and parental substance misuse. Explanations for this situation are multifactorial (45, 102), including structural and systemic factors, policies that militate against an integrated holistic response, lack of resources, a lack of professional expertise and the need for a theoretical underpinning to bring together the contribution of different professionals from police to family justice practitioners. In England, as austerity has bitten harder, increasing poverty and family vulnerability have coincided with a prolonged decline in substance misuse and DA services for victims, perpetrators and children with a significant lack of funding (105–107). At the same time as families became more isolated during Covid-19, there was a large rise in demand for help from agencies dealing with DA. On average calls and contacts to the National Domestic Abuse Helpline increased by 49% for the week commencing 6th April 2020 compared to pre-lockdown comparisons (108). Furthermore, heavy drinking and alcohol specific deaths rose significantly during the pandemic (109). Between March 2020 and March 2021 there was a 59% rise in the number of people who reported that they were drinking at increasing higher risk levels.

So, it is clear that despite the significant policy and legislative developments in recent years, DA remains a significant issue. Existing treatment and policy initiatives are not reducing DA and, of particular pertinence to this article, they are not addressing the co-occurring issue of substance misuse holistically. The significant costs of substance misuse^12^ and DA to society, as well as the current and future costs to the individual and family, make it imperative to find effective and sustainable interventions that can break the cycles of intergenerational harm associated with both parental substance misuse and DA. Nowhere is this more important than in the context of private and public law proceedings where there is a vital need to find alternative ways for the courts to respond to DA and parental substance misuse, and more specifically in an integrated way. Although IDACs offer promising potential, in their current format they do not appear to address the issue of co-occurrence of DA and substance misuse holistically. Given the association between these two problems, Family Drug and Alcohol Courts (FDACs) may provide a unique opportunity of dealing with DA as well as substance misuse when care proceedings are initiated.

## What is the potential of FDACs to provide an integrated response to co-occurring domestic abuse and substance misuse in care proceedings?

FDAC was first introduced in England in 2008 to break cycles of harm linked to parental substance misuse. Adapted from an American model of family drug treatment courts, FDACs offer an alternative problem-solving approach to care proceedings, aiming to help parents with complex problems of substance misuse, mental health, DA, housing and relationship difficulties within the proceedings. Despite its name, the FDAC approach is built on the premise that substance misuse is never the only issue and that problems need to be tackled holistically. A key aim of FDAC is to rebuild parenting capacity and thereby facilitate safe family reunification. If parents are unable to successfully address their multiple problems within the child's timescales, alternative permanent homes for the children.

To date, FDAC evaluation outcomes are promising, particularly in respect of the higher rates of family reunification and substance misuse cessation following FDAC involvement compared to ordinary care proceedings (111). This finding is supported by a meta-analysis of international evidence (112) and cost benefit analysis of FDAC, that indicates savings to the public purse (113). These factors have led to investment by the Department for Education (114) and the Welsh Government in the expansion of FDACs. The former President of the Family Division (Head of Family Justice) endorsed FDAC (115) and the current President wishes to see an FDAC in all designated family judge areas in England and Wales (116). Parents appreciate FDACs for their fairness, transparent process, respect, and the intensive support they receive and their compassionate approach (117).

FDACs are underpinned by a theory known as therapeutic jurisprudence (TJ) (118, 119). Its central tenet is that the court itself can be an agent of therapeutic change, and that without exploring and responding to underlying causes of the problems, the role of the court is limited and unlikely to achieve lasting change. TJ draws on a wide range of disciplines to inform its approach, including psychology, motivational theory, social work, and restorative justice. It has led to a very distinctive set of practices that use the authority of the court to bring about change (120–122). Unlike ordinary care proceedings, FDAC judges meet fortnightly with the parent without lawyers being present to help motivate the parent, address problems holistically, and receive information on parental progress from the FDAC team and parents' key worker. These non-lawyer hearings comprise the therapeutic component of FDAC in the court setting and provide a crucial opportunity for parents to speak directly to the judge. The FDAC multidisciplinary team, which may include social workers, psychologists, substance misuse workers and domestic abuse specialists, is crucial to the delivery of the tailor-made help package to parents. This integrated package is developed jointly with Children's Services and the court. There is no equivalent to the FDAC team in ordinary care proceedings. Furthermore, unlike ordinary care proceedings, FDACs are non-adversarial and collaborative.

Although FDAC is not a dedicated DA intervention, profiling by FDAC providers based on self-report suggests that it is prevalent in the majority of FDAC cases. FDAC teams address DA in their holistic support offer, and as already noted, a number have specialist DA workers. The unique and integrated support offered by FDAC teams makes them well placed to deal with care proceedings where DA and substance misuse co-exist, as this kind of truly holistic approach is not currently available through interventions such as criminal courts or IDAC's. However, to date there are no descriptions of the way in which FDAC responds to DA, or of its impact. Nor is there evidence from American FDTCs on the prevalence of co-occurring substance misuse and DA, the interventions offered, or the outcomes for parents and their children^13^. Finally, there is a lack of evidence on whether FDTCs liaise and coordinate with criminal family violence courts as recommended (123). These are all issues that need to be tested and examined further in the future.

The arguments about the potential contribution of FDACs in addressing DA are therefore best made at present on the basis of the goal of family policy to keep families together wherever possible; the lack of effective DA interventions within child protection and family justice (98), especially for families who face structural inequalities and multiple needs (19). Additionally, particularly relevant to this article, the overlap in the treatment needs and recovery goals as between parental substance misuse and DA and superior outcomes of FDACs compared to ordinary care proceedings in relation to sustainable reunification and substance misuse cessation (111).

Parents with substance misuse and DA both face the problem of stigma, shame, and fear that disclosure will lead to child removal, with gender and maternal identities a key issue (124, 125). Both problems leave parents feeling powerless (126) with very low self-esteem and little sense of agency and self-belief (127). These problems are exacerbated by societal stereotypes of blame and censure for these mothers. If parents are to be able to address both these issues, they need help to understand and address earlier patterns of behavior. To this end, the FDAC multidisciplinary team provides intensive support, links parents to community services, and helps with practical problems during the court process. Because of the evidence on the impact of early childhood adversity, FDACs offer a trauma informed treatment approach as a key element in recovery. This approach is particularly relevant for parents going through care proceedings because their own life experiences frequently make it particularly difficult to engage with professionals, whilst the adversarial nature of the proceedings further creates mistrust and antagonism (117).

## Looking to the future: Challenges and opportunities

FDAC is not a magic bullet, but it has the potential to address the co-occurring problems of DA and parental substance misuse in an integrated way at a crisis point. Moreover, whilst until recently there has been no robust way of evaluating judicial therapeutic behavior and interactional style in the courtroom to see how far it helps explain outcomes, a standardized tool is now available that will enable researchers to measure the components (122). Although the tool is limited to the interactional style of judges and has been developed in a criminal justice context, it provides an important way forward to test the components and values of TJ empirically. Additionally, a Protocol to undertake a Campbell collaboration systematic review has been funded to examine FDTCs' parental psychosocial and legal outcomes beyond parental substance misuse and child reunification (128). Previous lack of such reviews has been a significant limitation in being able to evaluate their potential contribution.

Despite the potential of FDACs to provide an integrated approach to DA and substance misuse in care proceedings, the problems are so widespread that it is care proceedings in general which need to change to provide the compassionate and more effective problem-solving approach found in FDAC. The same point has been made in the US context and Australia (129). For this reason, international TJ practitioners and theorists are working toward mainstreaming the approach into criminal law (120). In England and Wales, the Independent Review of Children's Social Care has recommended that the compassionate investigative approach found in FDAC should be extended to ordinary care proceedings in the family court (51). To this end, a new Care Proceedings Reform Group has been appointed by the President of the Family Division to make recommendations on how this can be achieved.

Changing the culture of the family courts would be a major step forward. But it would not be enough. Family courts also need to offer intensive help during proceedings and to ensure that such help continues to be available after proceedings end, given the fragility of reunification when parental substance misuse is involved. Moreover, private and public law care proceedings do not take place in a vacuum. The ever harsher macro-economic and social climate places particular burdens on disadvantaged families and repeated calls for major investment in social care reform have not been addressed. The possibility of legal aid for litigants in person in private law family proceedings remains unresolved, more than 2 years after it was first proposed. At a local level in the field of social care, research has shown that there is an inverse relationship between the amount of support provided by local authorities and the level of deprivation. The more disadvantaged the area, the less support is available (130). Furthermore, continued cuts to DA services and refuge space mean that accessing support, particularly when criminal justice intervention is not desired by the victim-survivor, is not a guarantee (69). There are also greater restrictions on available refuge space for victim-survivors who misuse substances, as many DA refuges have zero tolerance policies for alcohol and drug-taking (69). Despite this gloomy appraisal of obstacles to reform, there are also clear opportunities. Introducing multidisciplinary teams based in local schools and community settings, including DA and mental health workers, may make it easier for parents to access holistic support in a non-stigmatizing way, as proposed by the Independent Review of Children's Social Care (51). How far this proposed service will help reduce the number of cases coming before the family court will not be known for many years and will depend on the skill of the workforce, the investment in the scheme and the supply of services and the wider macro-economic context.

What is clear however, is that at present there is a lack of evaluated effective services for children and parents with DA and substance misuse difficulties, either singly or in combination, as well as a serious gap in availability. There is also a significant data deficit. As already noted, at the present time it is not possible to say how many children are affected by co-occurring DA and substance misuse in care proceedings or in private law proceedings. There need to be more sophisticated ways of studying the scale and pattern of domestic abuse when co-existing with parental substance misuse than are currently available. More attention needs to be paid to the response to DA and substance misuse in public law proceedings and the family court remains insufficiently integrated into policy around crime. Ultimately, more family support is vital, before, during and after proceedings. Recovery from DA, mental health problems and substance misuse, whether singly or in combination, require highly skilled and intensive support over time. We need to better understand the ingredients of such support and what works for which parents and why. FDACs provide one such opportunity to examine this issue.

## Discussion and implications

This review has examined the inter-relationship between co-occurring substance misuse and DA and their impacts on children. It has considered the availability of integrated family interventions and paid particular attention to the family court and the role it can play in addressing the inter-relationship between substance misuse and DA, including discussion of the potential of FDACs to deliver a holistic intervention. Finally, it has examined how far there is a coherent cross-cutting criminal and family legal and policy framework to underpin practice and to overcome the wellestablished criticism by Hester (82) that responses to DA have been fragmented and delivered in silos. With little change in this strategy since Hester's time of writing, her work remains pertinent.

There are three key findings of this review that address our five main research questions. Each has associated implications, which we shall summarize in turn.

1. A first key finding of this review is the lack of information on the prevalence of co-occurring substance misuse and DA at national population level and within the child protection and family justice arenas. It is simply not possible to report on the scale and pattern of co-occurrence, despite the clear evidence that co-occurrence of these two problems is more harmful to children than when only one issue is present. This is a data deficit issue, but it also has profound implications for designing tailor-made interventions that address both issues seamlessly and for tracking child and parent outcomes to know which interventions work and for whom. The data deficit also reflects the continued predominance of thinking in silos and seeing the two issues as separate rather than, as the evidence shows, overlapping and interconnected. This has significant safeguarding implications, as the potential harm to children increases when their parents are affected by both problems.
The challenge of addressing the data deficit is significant in child protection and the family court because at present there is a more basic problem. The databases do not flag DA or parental substance misuse. This is therefore a first essential issue to address. While the importance of improving intelligence on DA in private law proceedings has been recognized (52, 53), attention to this issue is needed in public law proceedings. Despite this major obstacle, there are promising research approaches to exploring co-occurrence. Through methodologies such as latent class analysis, more nuanced understandings are emerging of the patterns of co-occurrence over time that can then be used to identify best practice, but also highlight areas of policy that need to be addressed.
2. Our second main finding relates to the alignment between criminal and family justice legislation. Notably the Domestic Abuse Act 2021 specifies clearly that the child is a victim, thereby aligning with the Adoption and Children Act 2002 legislation. This has laid new duties on children's services and housing authorities which have the potential to deal with critical safeguarding issues as part of a multi-agency response- it is a positive development. A new approach to intervention can also be seen in the pilot problem-solving criminal courts for substance misusers, DA perpetrators and female offenders that are currently being set up. This is also a very promising development. It is of course too early to establish whether they will deliver better outcomes than traditional approaches, but a critical issue will be how far they interconnect with the family court. These developments reflect the shift in thinking from “why doesn't she leave?” to “why doesn't he stop?” (91). One answer to that question must be to provide better intervention strategies than have been available up to now.
More broadly however, the issue of silos persists, and was acknowledged to be a major obstacle to reform as recently as 2018 in the Ministry of Justice Harm Panel Report. Issues of silo working are evident in SDVCs, with a lack of holistic support for co-occurring DA and substance misuse provided in this context (73). The Home Office consultation on domestic abuse strategy, welcome as it was, has also been criticized for giving insufficient attention to the role of the family court (91). As far more children and mothers are affected by DA in this context, irrespective of co-existing parental substance misuse, it seems vital to ensure that the role of the family court is fully recognized and to clearly specify areas of overlap and joint strategy.
3. Thirdly, beyond law and policy, the review has found that there is a lack of effective integrated family interventions for co-occurring substance misuse and DA and a lack of effective interventions for perpetrators for these issues. This is a discouraging finding in terms of efforts to reduce DA and substance misuse because the problems result in broken families, children being taken into care and concomitant significant societal impacts including the significant financial costs and wasted lives. There is a clear need to develop an early help service to reduce these risks and in this regard the recommendation by the Independent Review of Children's Social Care to develop family hubs makes sense.
However, there will always be families who will require the court to intervene and determine whether the children can safely remain with their parents or need permanent separation. It is within this context that FDACs may have potential to better address co-existing parental substance misuse and DA than traditional courts or IDACs. Although more work is needed to test the concept and explore the value of FDAC as a response to DA in the context of care proceedings, this review has identified that the conceptual and theoretical approach holds out considerable promise. However, this potential can only be achieved if there are fundamental changes to care proceedings and family court processes, as discussed previously.

In sum, Hester's (82) agenda for reform to break down the silos was ambitious, and the 2018 Ministry of Justice Panel report makes clear the extent and persistence of the challenges associated with this. They encompass culture, understandings and attitudes to victims and perpetrators, professional expertise, the availability of effective services and resources. This review has shown that there is far greater awareness of the problems than ten years ago. However, there are still significant obstacles to achieving holistic effective interventions, working across sectors, underpinned by coherent well aligned criminal and family justice policies to tackle concurrent substance and DA and thereby promote children's wellbeing.

Tackling some of these issues will be easier than others. Addressing the issue of the data deficit is more circumscribed than the wider issues of legal and policy reform and development of effective interventions and as such may be easier to tackle. As suggested earlier, a possible starting point would be to introduce into the national administrative child protection and court databases a flag to mark the presence of DA and drug and alcohol misuse. Developing consistent definitions would also be an important preliminary step. Further research using LCA profiling would be useful, especially if it can be used to track recovery patterns over time and can be linked to the broader socio-economic context, given that poverty, deprivation, and housing problems are consistently linked to these problems. With regard to best practice, a systematic review of international evidence of co-occurrence would be of value. In relation to the role of the family court, more work needs to be done to shift from a theoretical argument in favor of FDACs to assist children and parents affected by DA, both as a single and co-occurring issue. Developing pilots with this goal in mind will be important, to see if they can produce better outcomes than ordinary child protection proceedings and enable families to stay together safely and sustainably. However, it is recognized that the challenges of aligning policy and practice across the criminal and family justice sector and between private and public law are considerable and there are no easy answers as to how they can be addressed.

## Conclusion

To conclude, this review has highlighted the well documented increased risk to mothers and children when parental substance misuse and DA co-exist. It has also shown what we know, where the gaps in our knowledge are, and has identified some of the challenges in addressing them. Whilst the focus has been on an English context, at least three key problems associated with how this issue is currently addressed in England and Wales, are relevant to many other jurisdictions across the globe. The first issue relates to the emphasis on criminal justice responses as the key way to prevent and reduce DA. There has been a flurry in legislative changes which intend to protect victim-survivors of DA, including the Domestic Abuse Act (2021) and the criminalization of coercive control (Serious Crime Act, 2015). However, in spite of this emphasis on criminal law and criminal justice interventions (81, 131), the prevalence and associated costs of DA remain consistently high. The jury is still out regarding whether legislative changes have helped to keep women and children safe or whether more law is the answer in relation to violence against women and girls more broadly (132). The statistics and evidence suggest that existing legislation has done relatively little to help women and children who experience DA (72).

The second issue relates to siloed approaches to responding to DA, substance misuse and mental health in spite of their clear overlaps. Hester (82) suggests that organizations working in the field of domestic violence, child protection and child contact are all essentially working from “different planets” and are difficult to bring together in a cohesive and coordinated way. Furthermore, treatment services are also often delivered in silos, despite the wealth of evidence highlighting the association between DA and parental substance misuse (102). An issue associated with this siloed working is a data deficit, resulting in a lack of a clear understanding of the scale and pattern of DA when co-existing with parental substance misuse. Thirdly and relatedly is a lack of a whole systems, holistic approaches to dealing with DA and parental substance misuse. Hester (82) suggests that a systematic change is required, emphasizing a unified, coordinated approach to dealing with DA.

This review has highlighted that one such way of enabling holistic, joined up approaches to responding to DA and substance misuse, which move away from focussing solely on legislative response, are FDACs. Evidence has highlighted their positive contribution to reunification and, with rigorous description and evaluation, could help extend the range of interventions designed to keep families together, challenging the view that courts are only sites of last resort.

In conclusion, the picture is very mixed as to the progress made in tackling co-occurrence of substance misuse and DA. What is clearer is where the gaps in our understanding are greatest and that the reform agenda is wide-ranging, ambitious and challenging.

## Author contributions

JH and CB took equal responsibility for the production of the review. JH contributed her expertise in family law, care proceedings, parental substance misuse, the idea for the review, and family drug courts. CB brought her expertise in domestic abuse, criminal law, and its impacts. Both authors contributed to the article and approved the submitted version.

## Funding

Lancaster University has agreed to cover the costs of open access publication.

## Conflict of interest

The authors declare that the research was conducted in the absence of any commercial or financial relationships that could be construed as a potential conflict of interest.

## Publisher's note

All claims expressed in this article are solely those of the authors and do not necessarily represent those of their affiliated organizations, or those of the publisher, the editors and the reviewers. Any product that may be evaluated in this article, or claim that may be made by its manufacturer, is not guaranteed or endorsed by the publisher.

## Footnotes

^1^In the USA family drug treatment courts are also known as family drug courts and family treatment drug courts.

^2^As of 2022, questions on coercive and controlling behavior will be included in the survey. https://www.ons.gov.uk/peoplepopulationandcommunity/crimeandjustice/articles/redevelopmentofdomesticabusestatistics/researchupdatenovember2021.

^3^8.1% of the 1.3 million women reporting on their most recent episode of DA for the Crime Survey for England and Wales, stated that they were under the influence of alcohol, with lower rates (1.7%) under the influence of drugs (46).

^4^Under s.17 of the Children Act 1989, children are deemed to be children in need if [a] they are unlikely to achieve or maintain or to have the opportunity to achieve or maintain a reasonable standard of health or development without provision of services from the Local Authority; [b] their health or development is likely to be significantly impaired, or further impaired, without the provision of services from the Local Authority; [c] they have a disability.

^5^Cafcass: the Children and Family Court Advisory and Support Service.

^6^s.31 (2) Children Act 1989.

^7^s.75A and s.75B Domestic Abuse Act 2021.

^8^Part 4, Domestic Abuse Act, 2021.

^9^Tier one local authorities include county councils, the Greater London Authority, and metropolitan and unitary authorities.

^10^s. 120 Adoption and Children Act 2002.

^11^Defined as ‘conflicts that occur between parents/carers that are frequent, intense and poorly resolved' but fall below the level of domestic abuse.

^12^Illegal drug use costs society approximately £20 billion per year (110) https://www.gov.uk/government/publications/review-of-drugs-phase-one-report/review-of-drugs-summary#part-one—the-illicit-drugs-market.

^13^Personal communication. Caroline S Cooper. Key Informant.
